# Glucose-to-potassium ratio as a predictor for early post-traumatic epilepsy: a retrospective cohort study

**DOI:** 10.3389/fneur.2025.1555328

**Published:** 2025-04-15

**Authors:** Jiayin Wang, Shukai Wu, Jiani Chen, Jinzhong Huang, Dankui Zhang

**Affiliations:** Department of Neurosurgery, Second Affiliated Hospital of Fujian Medical University, Quanzhou, China

**Keywords:** traumatic brain injury, early post-traumatic epilepsy, glucose-to-potassium ratio, biomarker, risk prediction, Kaplan-Meier curve analysis

## Abstract

**Background:**

Post-traumatic epilepsy (PTE) is a common complication following traumatic brain injury (TBI). Early PTE refers to the appearance of seizure symptoms within 7 days of the injury. The glucose-to-potassium ratio (GPR) has emerged as a potential biomarker for predicting Early PTE risk. This study aimed to evaluate the association between GPR and the risk of Early PTE, and to assess the predictive value of GPR through various analyses.

**Methods:**

A total of 2,049 TBI patients were included in the analysis, with the GPR evaluated both as a continuous and categorical variable. Logistic regression, trend tests, and Kaplan-Meier (KM) curve analyses were performed to assess the relationship between GPR and Early PTE. Subgroup analyses were conducted to explore potential effect modifiers, and restricted cubic spline (RCS) analyses were used to examine non-linear associations. Adjustments were made for demographic, clinical, and biochemical factors.

**Results:**

The GPR demonstrated a significant non-linear association with Early PTE risk, with a turning point at GPR = 2.835. Patients with a GPR > 2.835 exhibited a higher risk of epilepsy, as indicated by KM curve analysis (*P* < 0.0001). Logistic regression analysis revealed that GPR was an independent predictor of Early PTE in both unadjusted and adjusted models. In the fully adjusted model, GPR remained significantly associated with early epilepsy (OR: 1.499, 95% CI: 1.188–1.891, *P* < 0.001). Subgroup analyses identified gender, hypertension, and diabetes as significant effect modifiers. Trend tests revealed a dose-response relationship between GPR quartiles and epilepsy risk, with the highest quartile showing a significantly higher risk in both unadjusted and partially adjusted models (*P* = 0.017).

**Conclusions:**

The GPR is a robust and independent predictor of Early PTE, with higher GPR levels strongly associated with an increased risk of epilepsy. The non-linear relationship and variations across subgroups underscore the clinical utility of GPR in risk stratification and personalized management of TBI patients.

## 1 Introduction

Traumatic brain injury (TBI) is a major global health concern and one of the leading causes of death and disability, particularly among younger populations (1). The consequences of TBI extend beyond the acute injury, often leading to long-term complications such as post-traumatic epilepsy (PTE), which accounts for ~5% of all epilepsy cases and up to 20% of structural epilepsies (2). Early PTE, as an early manifestation of epilepsy, significantly impairs neurological recovery and is associated with poorer quality of life, increased healthcare costs, and worse functional outcomes (3). Despite advances in understanding TBI and PTE, the mechanisms underlying epileptogenesis—the process by which normal brain tissue becomes prone to seizures—remain poorly understood (4). Early identification of patients at risk for Early PTE is crucial for effective management, yet existing clinical tools and imaging biomarkers provide limited predictive power.

Biomarkers have emerged as valuable tools for predicting outcomes in TBI and guiding clinical decision-making. Recent studies have highlighted the glucose-to-potassium ratio (GPR) as a promising biomarker due to its simplicity, cost-effectiveness, and association with neurological outcomes (5, 6). GPR is derived from routine blood tests and reflects metabolic changes induced by TBI, including glucose dysregulation and electrolyte imbalances (7). Elevated GPR has been linked to increased release of excitatory neurotransmitters and disrupted neuronal ion balance, both of which are implicated in epileptogenesis (2). Compared to traditional biomarkers such as S100B and glial fibrillary acidic protein (GFAP), GPR offers practical advantages for routine clinical use, making it a viable candidate for stratifying patients at risk of Early PTE (5).

Although the relationship between GPR and mortality in TBI has been explored, its role in predicting Early PTE remains under-investigated. This study aims to evaluate GPR as an independent predictor of Early PTE, assess its non-linear associations with epilepsy risk, and explore its performance across various subgroups. By leveraging a large TBI cohort and robust statistical models, this research seeks to validate the clinical utility of GPR for early risk stratification in TBI patients. Establishing GPR as a predictive biomarker may improve targeted interventions. The clinicians can calculate the GPR value based on the results of the first blood glucose and blood potassium detection on admission, so as to evaluate the probability of Early PTE and provide early treatment for epilepsy prevention, and reducing the burden of Early PTE and enhancing patient outcomes.

## 2 Method

### 2.1 Study population

This retrospective cohort study included 2,049 patients diagnosed with TBI and treated at the Second Affiliated Hospital of Fujian Medical University between September 1, 2014, and September 1, 2024. The inclusion criteria were as follows: (1) Patients diagnosed with TBI and admitted to the hospital within 24 h. (2) Age ≥ 18 years. (3) Availability of sufficient clinical and biochemical data, including glucose and potassium levels, within 24 h of admission. (4) Definition of seizures occurring within 7 days following TBI. (5) Patients who received comprehensive treatment and follow-up in the hospital. On the other side, the exclusion criteria included: (1) A history of epilepsy, or other pre-existing neurological disorders prior to TBI. (2) Presence of significant comorbidities, such as malignancies, severe organ dysfunction (e.g., liver, kidney, or cardiovascular failure), or severe systemic diseases (e.g., uncontrolled hypertension, respiratory failure). (3) Patients with incomplete medical records or missing laboratory data. (4) Admission delayed beyond 24 h after the initial injury. (5) A hospital stay of fewer than 48 h. (6) Severe associated injuries, such as polytrauma or extracranial conditions that could affect prognosis. (7) Patients receiving immunosuppressive or antiepileptic treatment prior to TBI.

### 2.2 Ethical statement

The study was approved by the Institutional Ethics Committee of the Second Affiliated Hospital of Fujian Medical University, in accordance with the Declaration of Helsinki (2023, Ethics Review No. 287). Written informed consent was waived due to the retrospective nature of the study. All patient data were anonymized prior to analysis to ensure confidentiality.

### 2.3 Data collection

Baseline demographic and clinical characteristics, including age, sex, Glasgow Coma Scale (GCS) scores, and hospital stay duration, were collected from medical records. Laboratory values such as glucose, potassium, white blood cell (WBC) count, hemoglobin, and platelets were obtained within 24 hours of admission. The GPR was calculated as serum glucose (mmol/L) divided by serum potassium (mmol/L) (8). The definition of Early PTE as epileptic seizures occurring within 7 days after TBI, manifested as clinically evident seizures (focal or generalized) or EEG-documented epileptiform discharges (e.g., focal spike-and-wave complexes or epileptic electrical status) (9).

### 2.4 Statistical analysis

Continuous variables were expressed as mean ± standard deviation (SD) and compared using the Student's *t-*test or Mann-Whitney U test, depending on normality. Categorical variables were analyzed using chi-squared or Fisher's exact tests, as appropriate. Logistic regression was performed to assess the independent association between GPR and Early PTE, with results expressed as odds ratio (OR) and 95% confidence intervals (CI). Subgroup and trend analyses were conducted to explore interactions between GPR and potential risk modifiers such as gender, hypertension and diabetes (10–12). Kaplan-Meier (KM) curves were used to evaluate the cumulative incidence of epilepsy based on dichotomized GPR (GPR > 2.835 vs. ≤ 2.835), and differences were assessed using the log-rank test. Restricted cubic spline (RCS) models were employed to evaluate the non-linear association between GPR and early epilepsy risk. Statistical significance was set at *P* < 0.05. Analyses were performed using SPSS version 26.0 and R software (version 4.2.2).

## 3 Results

**Figure 1:**
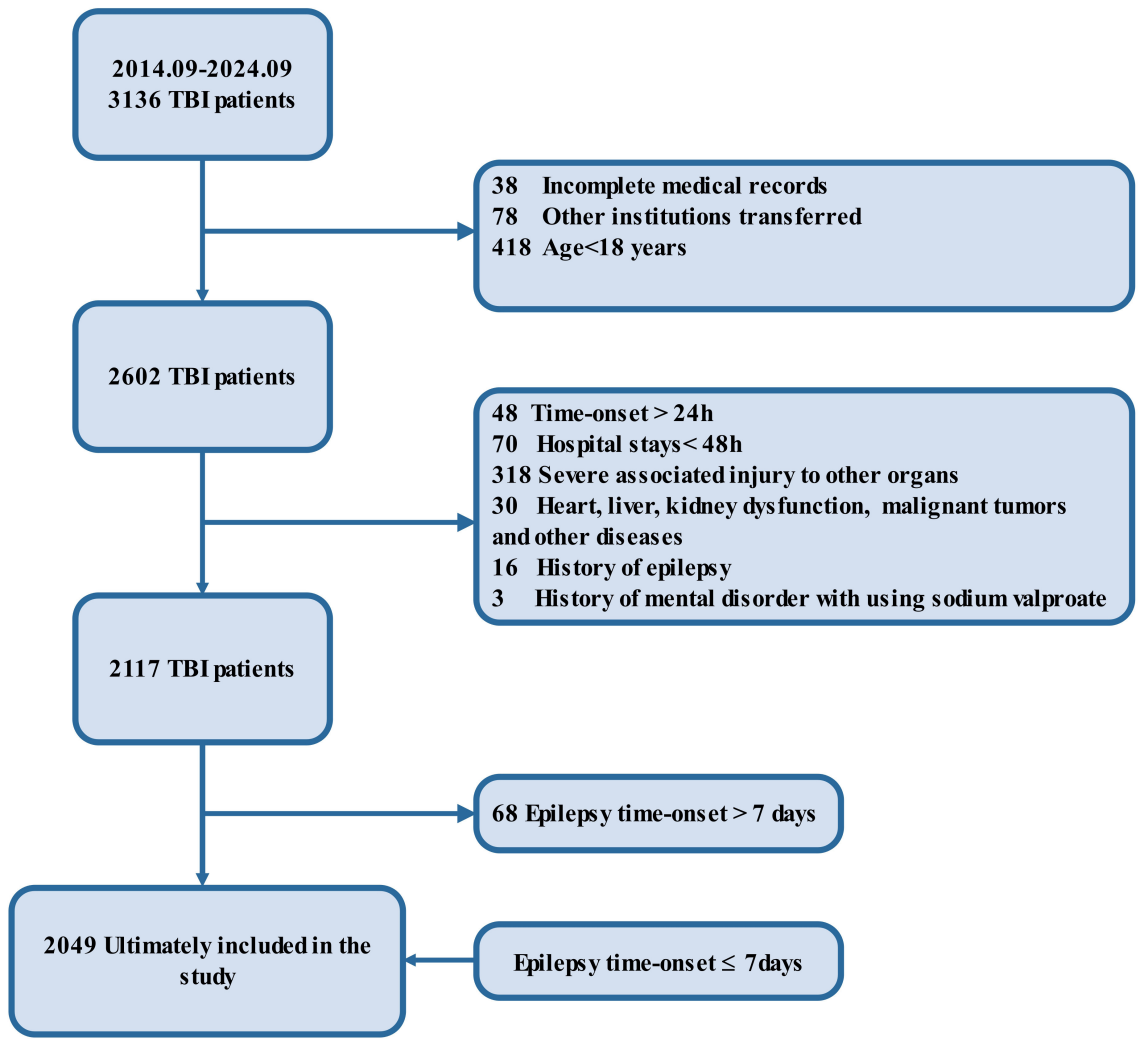
Flow diagram. The flowchart outlines the selection criteria and patient exclusions for the study on traumatic brain injury (TBI). Initially, 3,136 patients with TBI were identified between September 2014 and September 2024. After excluding 1,034 patients due to incomplete records, age below 18, or transfer to other institutions, 2,602 patients remained. Further exclusions based on late time-onset (>24 h), short hospital stays (< 48 h), severe associated injuries, organ dysfunction, malignancies, epilepsy history, and mental health disorders reduced the cohort to 2,117 patients. Of these, 68 patients were excluded due to epilepsy onset after 7 days, leaving 2,049 patients included in the final analysis with epilepsy onset ≤ 7 days.

A total of 3,136 patients with TBI were initially screened for inclusion in the study between September 1, 2014, and September 1, 2024. After excluding 1,034 patients due to incomplete medical records, age under 18 years, or transfers from other institutions, 2,602 patients remained eligible for further evaluation. Additional exclusions were made for 485 patients based on late treatment onset (>24 h), short hospital stays (< 48 h), severe associated injuries, or organ dysfunction. Furthermore, patients with a history of epilepsy, mental health disorders requiring sodium valproate, or other significant comorbidities were excluded, resulting in a final cohort of 2,117 patients. To ensure the focus remained on early epilepsy outcomes, 68 patients with epilepsy onset beyond 7 days post-TBI were further excluded. Ultimately, 2,049 patients met all inclusion criteria and were included in the study, with early epilepsy onset occurring within 7 days of cerebral trauma (3) (Figure 1).

### 3.1 Baseline demographic and clinical characteristics

The study population revealed significant differences between patients with and without early epilepsy, highlighting key risk factors for Early PTE. Patients who developed early epilepsy were significantly older than those without epilepsy, both before and after propensity score matching (PSM, the match is performed 1:1) (*P* < 0.001). Epilepsy patients also had longer hospital stays, with an average stay of 41.15 days before matching and 35.28 days after matching, compared to 22.23 and 20.53 days in non-epilepsy patients, respectively (*P* < 0.003). GCS scores were lower in epilepsy patients, indicating more severe injuries. Additionally, the time from TBI onset to hospital admission was significantly shorter in epilepsy patients, reflecting the acute nature of their condition (*P* < 0.05).

Laboratory findings revealed key differences between the groups. Early PTE patients had higher GPR, both before (*P* < 0.001) and after PSM (*P* = 0.003), highlighting its potential as a predictive marker. Inflammatory markers, such as WBC counts, were elevated in epilepsy patients, while platelet counts and hemoglobin levels were lower, suggesting possible systemic responses to injury. Hypertension was more common in the epilepsy group, and epilepsy patients were more likely to undergo surgical interventions, even after PSM (*P* = 0.005). These results, summarized in Table 1.

**Table 1:** Demography of the study population.

**Variable**	**Before PSM**			**P-value**	**After PSM**			**P-value**
	**Overall**	**Non-epilepsy**	**Epilepsy**		**Overall**	**Non-epilepsy**	**Epilepsy**	
	***N** =* **2,049**	***N** =* **1,977**	***N** =* **72**		***N** =* **139**	***N** =* **70**	***N** =* **69**	
Age, mean (SD)	55.89 (17.03)	55.65 (16.88)	62.46 (19.55)	0.005	74.81 (19.00)	87.03 (5.55)	62.42 (19.81)	< 0.001
GCS, mean (SD)	12.92 (3.56)	12.98 (3.51)	11.47 (4.60)	0.007	12.36 (4.00)	13.00 (3.34)	11.71 (4.51)	0.058
Time_onset, mean (SD)	392.33 (2,326.54)	406.14 (2,367.40)	13.18 (12.42)	< 0.001	50.43 (209.96)	86.90 (292.04)	13.43 (12.63)	0.039
Hospital_stays, mean (SD)	22.90 (24.55)	22.23 (22.90)	41.15 (49.41)	0.002	27.85 (29.95)	20.53 (26.13)	35.28 (31.88)	0.003
PLT, mean (SD)	208.35 (86.76)	209.29 (87.35)	182.57 (63.76)	< 0.001	173.54 (63.57)	166.06 (63.09)	181.13 (63.61)	0.163
Hb, mean (SD)	124.12 (21.48)	124.26 (21.35)	120.38 (24.61)	0.191	114.99 (23.13)	109.70 (20.95)	120.36 (24.14)	0.006
RBC, mean (SD)	4.09 (0.73)	4.10 (0.73)	3.94 (0.76)	0.090	3.78 (0.71)	3.64 (0.63)	3.92 (0.76)	0.018
Monocyte, mean (SD)	0.61 (0.30)	0.61 (0.30)	0.63 (0.35)	0.544	0.59 (0.30)	0.55 (0.21)	0.63 (0.36)	0.112
Lymphocyte, mean (SD)	1.35 (0.66)	1.36 (0.66)	1.08 (0.57)	< 0.001	1.12 (0.60)	1.15 (0.65)	1.08 (0.55)	0.507
WBC, mean (SD)	10.81 (4.64)	10.73 (4.59)	12.90 (5.26)	< 0.001	11.21 (4.74)	9.62 (3.51)	12.83 (5.28)	< 0.001
HDL, mean (SD)	1.18 (0.38)	1.18 (0.38)	1.25 (0.31)	0.076	1.24 (0.33)	1.22 (0.34)	1.25 (0.32)	0.538
LDL, mean (SD)	2.57 (0.86)	2.58 (0.86)	2.44 (0.80)	0.157	2.46 (0.84)	2.47 (0.86)	2.45 (0.82)	0.905
P, mean (SD)	0.97 (0.28)	0.97 (0.28)	0.88 (0.27)	0.007	0.89 (0.26)	0.89 (0.26)	0.88 (0.26)	0.950
Na, mean (SD)	139.18 (4.80)	139.18 (4.81)	139.22 (4.54)	0.935	139.15 (5.03)	139.02 (5.47)	139.29 (4.57)	0.751
Ca, mean (SD)	2.18 (0.15)	2.18 (0.15)	2.17 (0.15)	0.655	2.14 (0.14)	2.12 (0.14)	2.16 (0.15)	0.113
ALT, mean (SD)	29.04 (57.95)	28.99 (58.31)	30.35 (47.35)	0.813	23.42 (34.56)	18.74 (15.91)	28.18 (46.07)	0.111
AST, mean (SD)	33.14 (72.85)	32.86 (73.19)	40.93 (62.58)	0.289	33.21 (46.60)	27.75 (22.37)	38.75 (61.95)	0.169
CK, mean (SD)	385.36 (1,034.42)	381.29 (1,042.96)	497.24 (760.24)	0.214	350.76 (583.17)	221.46 (291.91)	481.94 (754.34)	0.009
Albumin, mean (SD)	39.29 (5.31)	39.27 (5.27)	39.94 (6.44)	0.387	38.52 (5.86)	37.25 (4.84)	39.80 (6.53)	0.010
Globulin, mean (SD)	25.67 (4.51)	25.62 (4.48)	27.08 (5.33)	0.024	26.92 (5.11)	26.79 (4.83)	27.06 (5.42)	0.759
APTT, mean (SD)	29.58 (6.07)	29.58 (6.09)	29.75 (5.46)	0.794	30.16 (5.72)	30.48 (5.96)	29.83 (5.49)	0.511
PT, mean (SD)	12.24 (1.97)	12.23 (1.98)	12.60 (1.77)	0.088	12.96 (3.48)	13.30 (4.56)	12.62 (1.79)	0.245
TT, mean (SD)	16.63 (4.80)	16.59 (4.47)	17.65 (10.35)	0.388	17.46 (8.65)	17.19 (6.29)	17.74 (10.56)	0.714
GPR, mean (SD)	1.79 (0.76)	1.77 (0.74)	2.36 (1.05)	< 0.001	2.10 (0.95)	1.87 (0.76)	2.34 (1.06)	0.003
Gender, *n* (*p*%)				0.706				0.313
Female	668.00 (32.60%)	646.00 (32.68%)	22.00 (30.56%)		48.00 (34.53%)	27.00 (38.57%)	21.00 (30.43%)	
Male	1,381.00 (67.40%)	1,331.00 (67.32%)	50.00 (69.44%)		91.00 (65.47%)	43.00 (61.43%)	48.00 (69.57%)	
Smoking, *n* (*p*%)				0.189				0.041
No	1,841.00 (89.85%)	1,773.00 (89.68%)	68.00 (94.44%)		135.00 (97.12%)	70.00 (100.00%)	65.00 (94.20%)	
Yes	208.00 (10.15%)	204.00 (10.32%)	4.00 (5.56%)		4.00 (2.88%)	0.00 (0.00%)	4.00 (5.80%)	
Drinking, *n* (*p*%)				0.098				0.151
No	1,886.00 (92.04%)	1,816.00 (91.86%)	70.00 (97.22%)		137.00 (98.56%)	70.00 (100.00%)	67.00 (97.10%)	
Yes	163.00 (7.96%)	161.00 (8.14%)	2.00 (2.78%)		2.00 (1.44%)	0.00 (0.00%)	2.00 (2.90%)	
Hypertension, *n* (*p*%)				0.014				0.014
No	1,533.00 (74.82%)	1,488.00 (75.27%)	45.00 (62.50%)		72.00 (51.80%)	29.00 (41.43%)	43.00 (62.32%)	
Yes	516.00 (25.18%)	489.00 (24.73%)	27.00 (37.50%)		67.00 (48.20%)	41.00 (58.57%)	26.00 (37.68%)	
Diabetes, *n* (*p*%)				0.886				0.518
No	1,748.00 (85.31%)	1,687.00 (85.33%)	61.00 (84.72%)		116.00 (83.45%)	57.00 (81.43%)	59.00 (85.51%)	
Yes	301.00 (14.69%)	290.00 (14.67%)	11.00 (15.28%)		23.00 (16.55%)	13.00 (18.57%)	10.00 (14.49%)	
Frontal_lobe, *n* (*p*%)				0.073				0.556
No	1,260.00 (61.49%)	1,223.00 (61.86%)	37.00 (51.39%)		76.00 (54.68%)	40.00 (57.14%)	36.00 (52.17%)	
Yes	789.00 (38.51%)	754.00 (38.14%)	35.00 (48.61%)		63.00 (45.32%)	30.00 (42.86%)	33.00 (47.83%)	
Temporal_lobe, *n* (*p*%)				0.039				0.080
No	1,498.00 (73.11%)	1,453.00 (73.50%)	45.00 (62.50%)		100.00 (71.94%)	55.00 (78.57%)	45.00 (65.22%)	
Yes	551.00 (26.89%)	524.00 (26.50%)	27.00 (37.50%)		39.00 (28.06%)	15.00 (21.43%)	24.00 (34.78%)	
Parietal_lobe, *n* (*p*%)				0.659				0.397
No	1,905.00 (92.97%)	1,839.00 (93.02%)	66.00 (91.67%)		126.00 (90.65%)	62.00 (88.57%)	64.00 (92.75%)	
Yes	144.00 (7.03%)	138.00 (6.98%)	6.00 (8.33%)		13.00 (9.35%)	8.00 (11.43%)	5.00 (7.25%)	
Occipital_lobe, *n* (*p*%)				0.827				0.661
No	2,000.00 (97.61%)	1,930.00 (97.62%)	70.00 (97.22%)		134.00 (96.40%)	67.00 (95.71%)	67.00 (97.10%)	
Yes	49.00 (2.39%)	47.00 (2.38%)	2.00 (2.78%)		5.00 (3.60%)	3.00 (4.29%)	2.00 (2.90%)	
Surgery, *n* (*p*%)				< 0.001				0.005
No	1,700.00 (82.97%)	1,652.00 (83.56%)	48.00 (66.67%)		105.00 (75.54%)	60.00 (85.71%)	45.00 (65.22%)	
Yes	349.00 (17.03%)	325.00 (16.44%)	24.00 (33.33%)		34.00 (24.46%)	10.00 (14.29%)	24.00 (34.78%)	
Death_in_hospital, n(*p*%) (*p*%)				0.565				0.151
No	1,965.00 (95.90%)	1,895.00 (95.85%)	70.00 (97.22%)		131.00 (94.24%)	64.00 (91.43%)	67.00 (97.10%)	
Yes	84.00 (4.10%)	82.00 (4.15%)	2.00 (2.78%)		8.00 (5.76%)	6.00 (8.57%)	2.00 (2.90%)	

GCS, Glasgow Coma Scale; PLT, Platelet; Hb, Hemoglobin; RBC, Red Blood Cell; WBC, White Blood Cell; HDL, High Density Lipoprotein; LDL, Low Density Lipoprotein; P, Phosphorus; Na, Natrium; Ca, Calcium; ALT, Alanine Transaminase; ASL, Aspartic Acid Transaminase; CK, Creatine Kinase; APTT, Activated Partial Thromboplastin Time; PT, Prothrombin Time; TT, Thrombin Time; GPR, Glucose-Potassium Ratio; PSM, Propensity Score Matching; SD, standard deviation.

### 3.2 Logistic regression analysis results

Logistic regression analysis identified several significant predictors of Early PTE both before and after PSM (Table 2). Before PSM, higher age (OR = 1.025, *P* = 0.001), longer hospital stays (OR = 1.014, *P* < 0.001), WBC counts (OR = 1.080, *P* < 0.001), and higher GPR (OR = 1.854, *P* < 0.001) were positively associated with increased epilepsy risk. Conversely, better GCS scores (OR = 0.909, *P* = 0.001), higher platelet counts (OR = 0.995, *P* = 0.006), and lower lymphocyte counts (OR = 0.434, *P* < 0.001) were protective factors.

**Table 2:** Logistic regression analysis result.

**Before PSM**					**After PSM**				
**Variable**	***P*** **-value**	**OR**	**Lower**	**Upper**	**Variable**	***P*** **-value**	**OR**	**Lower**	**Upper**
GCS	0.001	0.909	0.862	0.960	GCS	0.060	0.920	0.843	1.004
Age	0.001	1.025	1.010	1.040	Age	< 0.001	0.828	0.775	0.885
Time_onset	0.008	0.983	0.971	0.996	Time_onset	0.057	0.984	0.968	1.000
Hospital_stays	< 0.001	1.014	1.009	1.019	Hospital_stays	0.008	1.021	1.006	1.037
PLT	0.006	0.995	0.991	0.999	Hb	0.008	1.022	1.006	1.038
RBC	0.076	0.753	0.550	1.030	RBC	0.020	1.814	1.096	3.001
WBC	< 0.001	1.080	1.038	1.124	WBC	0.000	1.182	1.083	1.290
Lymphocyte	< 0.001	0.434	0.276	0.682	CK	0.021	1.001	1.000	1.003
P	0.006	0.293	0.122	0.703	Albumin	0.012	1.081	1.018	1.149
Globulin	0.007	1.069	1.018	1.122	Hypertension(1)	0.014	2.338	1.184	4.618
Hypertension(1)	0.016	0.548	0.336	0.892	Temporal_lobe(1)	0.082	0.511	0.240	1.089
Frontal_lobe(1)	0.075	0.652	0.407	1.044	Surgery(1)	0.006	0.313	0.136	0.719
Temporal_lobe(1)	0.041	0.601	0.369	0.979	GPR	0.005	1.793	1.194	2.692
Surgery(1)	< 0.001	0.393	0.238	0.651					
GPR	< 0.001	1.854	1.514	2.272					

GCS, Glasgow Coma Scale; PLT, Platelet; Hb, Hemoglobin; RBC, Red Blood Cell; WBC, White Blood Cell; P, Phosphorus; CK, Creatine Kinase; GPR, Glucose-Potassium Ratio; PSM, Propensity Score Matching; OR, Odds Ratio.

After PSM, age remained a significant predictor but showed an inverse association (OR = 0.828, *P* < 0.001). Longer hospital stays (OR = 1.021, *P* = 0.008), higher hemoglobin levels (OR = 1.022, *P* = 0.008), and elevated WBC counts (OR = 1.182, *P* < 0.001) were associated with increased epilepsy risk. Notably, GPR retained its significance after matching (OR = 1.793, *P* = 0.005), reaffirming its role as an independent predictor. Other significant factors included creatine kinase (CK) levels (OR = 1.001, *P* = 0.021), albumin (OR = 1.081, *P* = 0.012), and a history of hypertension (OR = 2.338, *P* = 0.014), whereas undergoing surgery was associated with a protective effect (OR = 0.313, *P* = 0.006).

### 3.3 Predictive value of GPR for early PTE

**Figure 2:**
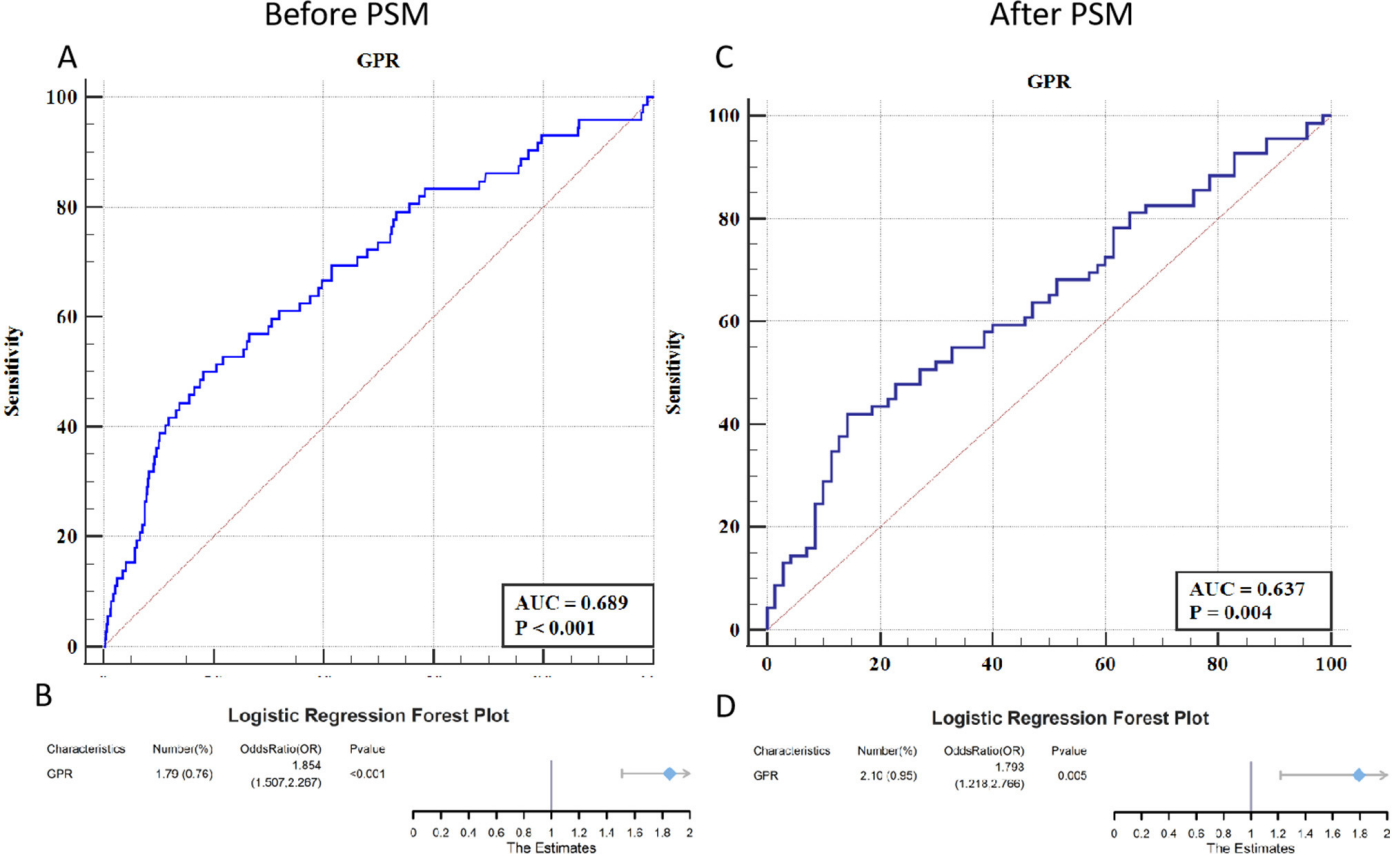
ROC curves and forest plots for the predictive value of GPR in early PTE. **(A)** The ROC curve before PSM shows an AUC of 0.689, indicating a moderate predictive capability of GPR for early PTE (*P* < 0.001). **(B)** The corresponding forest plot before PSM shows an OR of 1.854 (95% CI: 1.507–2.267, *P* < 0.001), highlighting GPR as a significant independent predictor. **(C)** The ROC curve after PSM yields an AUC of 0.637, reflecting a slightly lower predictive ability of GPR but still statistically significant (*P* = 0.004). **(D)** The forest plot after PSM shows an OR of 1.793 (95% CI: 1.218–2.766, *P* = 0.005), maintaining GPR's role as a significant predictor even after matching for confounders.

Figure 2 illustrates the predictive capability of the GPR for Early PTE through receiver operating characteristic (ROC) curves and logistic regression analysis. Before PSM, the ROC curve yielded an area under the curve (AUC) of 0.689 (*P* < 0.001), with a Youden index of 0.318 (sensitivity 50.00%, specificity 81.84%), indicating moderate predictive power for GPR. Logistic regression analysis further supported this finding, with an OR of 1.854 (95% CI: 1.507–2.267, *P* < 0.001), establishing GPR as a significant independent predictor of post-TBI epilepsy.

After PSM, the AUC for the ROC curve decreased slightly to 0.637 but remained statistically significant (*P* = 0.004), with a Youden index of 0.277 (sensitivity 42.03%, specificity 85.71%). Logistic regression analysis post-PSM demonstrated an OR of 1.793 (95% CI: 1.218–2.766, *P* = 0.005), confirming the robustness of GPR as a predictor even after adjusting for confounding factors. These results underscore the utility of GPR as a reliable biomarker for identifying TBI patients at risk of developing early epilepsy.

### 3.4 Non-linear association between GPR and early PTE risk

**Figure 3:**
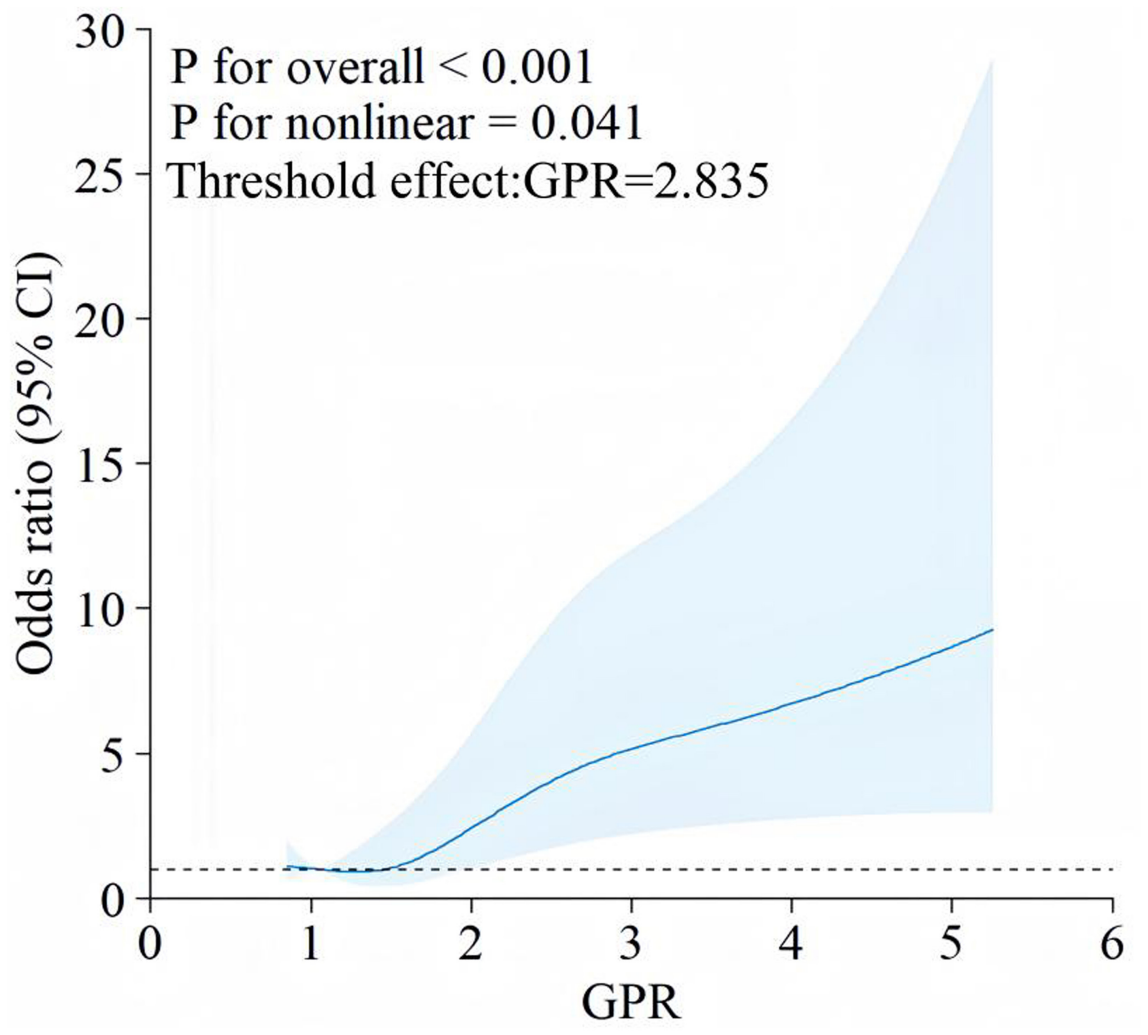
RCS analysis of the association between GPR and early PTE. This figure depicts a RCS plot demonstrating the relationship between the GPR and the OR of developing early PTE. The blue line represents the estimate 1d OR, with the shaded area indicating the 95% CI. The analysis shows a significant overall association (*P* < 0.001) and a non-linear relationship (*P* = 0.041) between GPR and early PTE.

**Figure 4:**
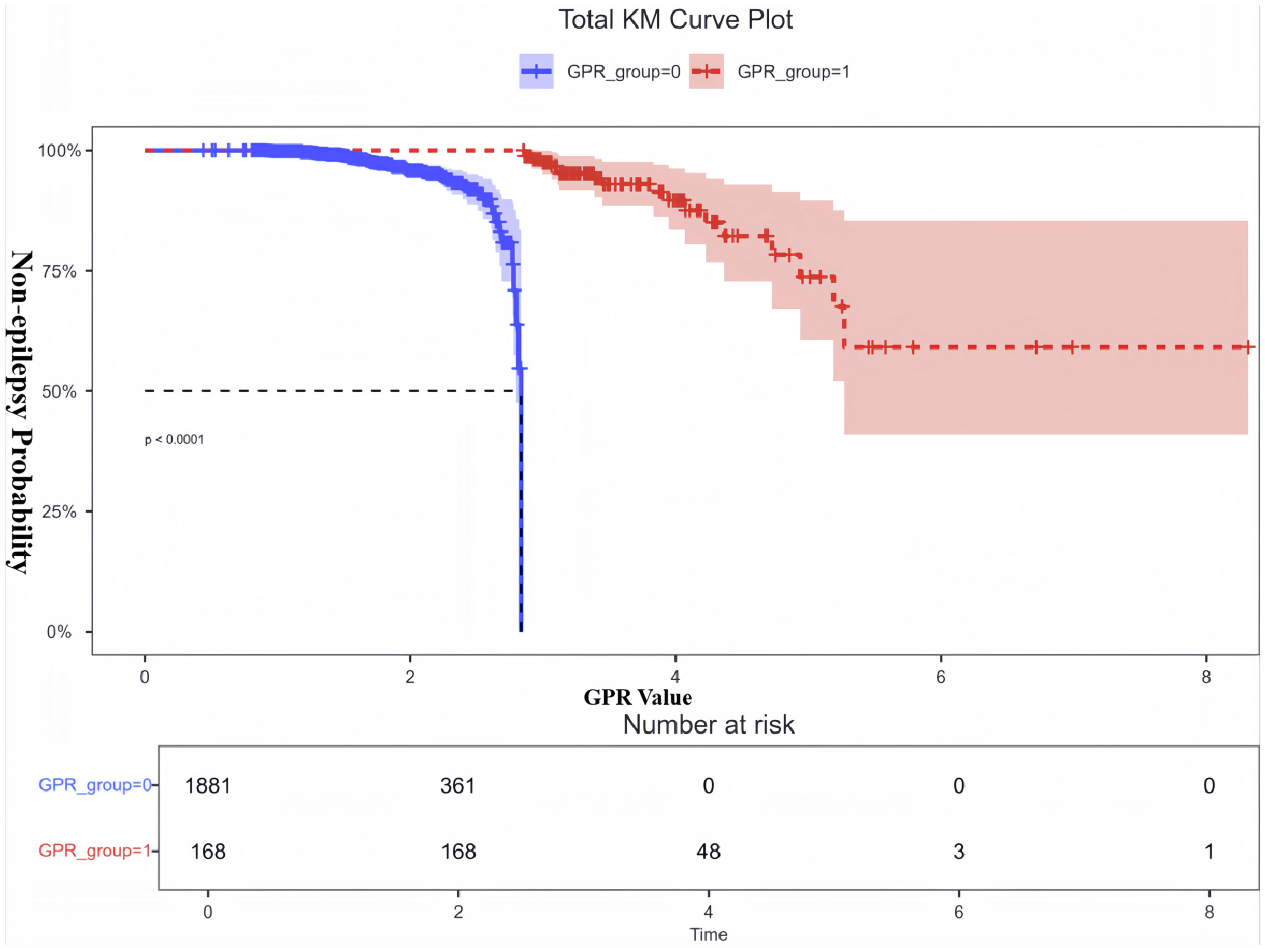
KM curve for non-epilepsy probability based on dichotomized GPR. This figure shows the KM analysis examining the probability of non-epilepsy in patients with dichotomized GPR. Patients were divided into two groups: GPR ≤ 2.835 (GPR_group = 0, blue) and GPR > 2.835 (GPR_group = 1, red). The *y*-axis represents the probability of remaining free from epilepsy, while the *x*-axis shows the time window derived from the actual GPR values. The results demonstrate a significant difference in non-epilepsy probability between the two groups (*P* < 0.0001). Patients with GPR > 2.835 had a markedly lower probability of remaining epilepsy-free over time, as indicated by the red dashed curve. The shaded areas represent the 95%CI for each group. The number at risk at each time point is displayed below the plot. These findings highlight the prognostic value of GPR in identifying patients at high risk for early PTE.

Figure 3 illustrates the non-linear relationship between GPR and the odds of developing Early PTE, as shown by RCS analysis. The overall association between GPR and Early PTE risk was statistically significant (*P* < 0.001), with evidence of non-linearity (*P* = 0.041). Threshold effect analysis identified a non-linear relationship, with a turning point at GPR = 2.835. The OR for epilepsy increased gradually as GPR exceeded 2.835, with a more pronounced rise at higher GPR values. Patients were therefore classified into two groups: GPR ≤ 2.835 (GPR_group = 0) and GPR > 2.835 (GPR_group = 1). KM curve analysis of the cumulative probability of remaining free from early epilepsy in patients with dichotomized GPR (Figure 4) revealed a significant difference between the two groups (*P* < 0.0001). Patients in the GPR > 2.835 group showed a markedly lower probability of avoiding epilepsy over time. The KM curves highlight that patients with GPR > 2.835 (red group) experienced a more rapid decline in the probability of remaining free from epilepsy compared to those with GPR ≤ 2.835 (blue group).

### 3.5 GPR multiple model regression analysis

For validating GPR as an independent risk factor for predicting Early PTE, this study constructed three models. Model-1 was unadjusted for any variables. Model-2 adjusted for general demographic and clinical covariates, including gender, age, smoking, drinking, hypertension, and diabetes. Model-3 further adjusted for covariates with *P* < 0.05 identified in the logistic regression analysis. The multiple model regression analysis of GPR demonstrated its robust association with Early PTE across different adjustment levels (Table 3).

**Table 3:** GPR multiple model regression analysis.

**Exposure**	**Model-1**		**Model-2**		**Model-3**	
	**OR (95%CI)**	***P*** **-value**	**OR (95%CI)**	***P*** **-value**	**OR (95%CI)**	***P*** **-value**
GPR	1.854 (1.514, 2.272)	< 0.001	2.010 (1.599, 2.526)	< 0.001	1.499 (1.188, 1.891)	< 0.001

Model-1: non-adjusted.

Model-2: adjust for: Gender, Age, Smoking, Drinking, Hypertension, Diabetes.

Model-3: adjust for: GCS, Age, PLT, RBC, Lymphocyte, WBC, P, Globulin, Hypertension, Frontal_lobe, Temporal_lobe, Surgery.

GCS, Glasgow Coma Scale; PLT, Platelet; RBC, Red Blood Cell; WBC, White Blood Cell; P, Phosphorus; GPR, Glucose-Potassium Ratio; OR, Odds Ratio; CI, confidence interval.

In the unadjusted model (Model-1), GPR showed a strong and significant association with early epilepsy risk, with an OR of 1.854 (95% CI: 1.514–2.272, *P* < 0.001). After adjusting for demographic and lifestyle factors such as gender, age, smoking, drinking, hypertension, and diabetes (Model-2), the association strengthened further (OR: 2.010, 95% CI: 1.599–2.526, *P* < 0.001). In the fully adjusted model (Model-3), which included clinical and biochemical variables such as GCS, platelet count (PLT), red blood cell count (RBC), lymphocyte count, WBC, phosphorus, globulin, hypertension, and brain regions affected (frontal and temporal lobes), the association remained significant but was slightly attenuated (OR: 1.499, 95% CI: 1.188–1.891, *P* < 0.001). From that, it confirm that the GPR is an independent predictor of early PTE, even after controlling for a comprehensive set of confounding factors.

### 3.6 Subgroup analysis of GPR and early PTE

**Figure 5:**
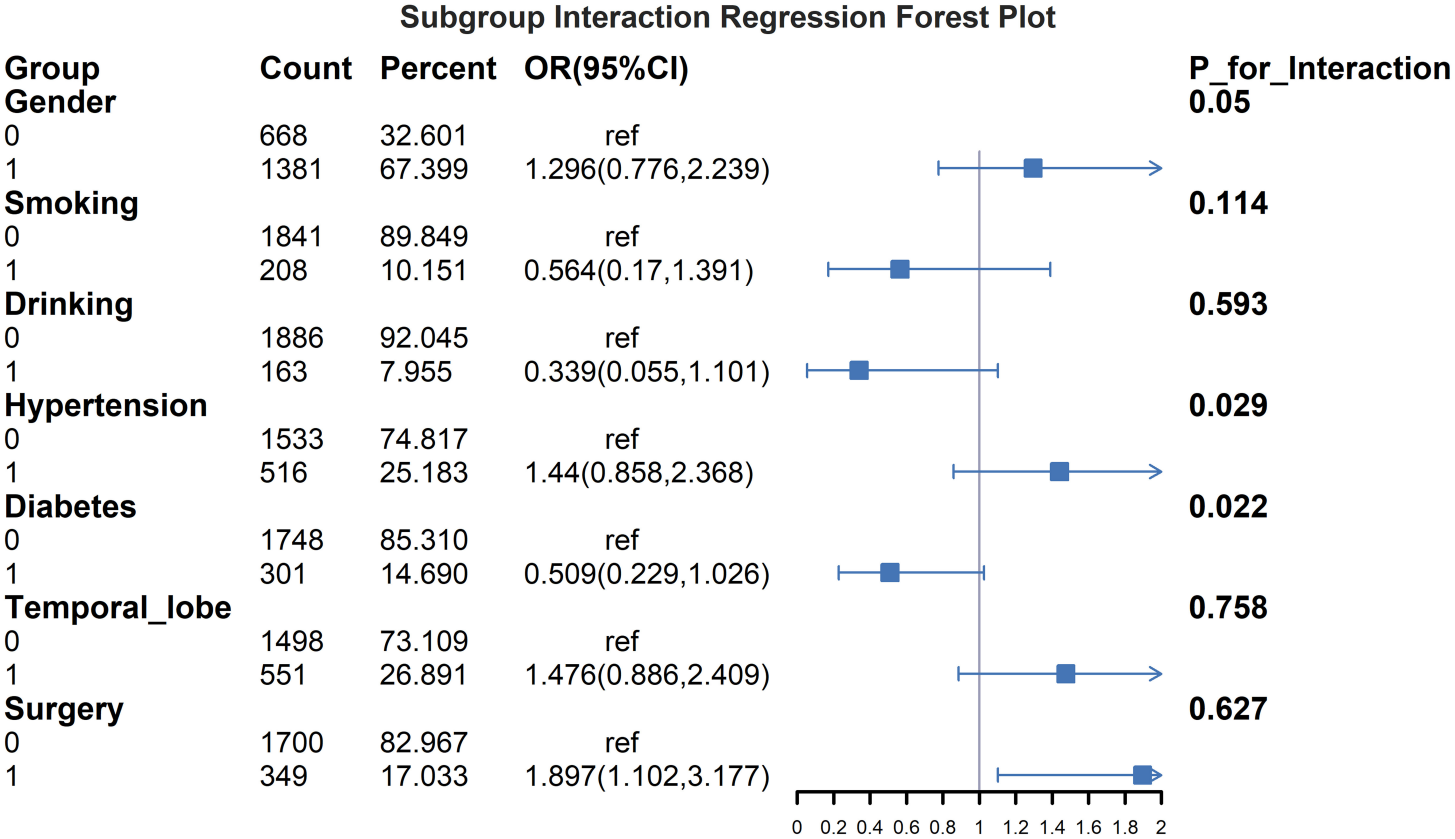
Subgroup interaction regression forest plot for GPR and early PTE. This figure illustrates the interaction effects of various subgroups on the association between the GPR and the risk of early PTE. OR with 95%CI are displayed for each subgroup, including gender, smoking, drinking, hypertension, diabetes, temporal lobe involvement, and surgery. The results highlight significant interactions for gender (P for interaction = 0.05), hypertension (*P* for interaction = 0.029) and diabetes (*P* for interaction = 0.022). Patients with male, hypertension and those without diabetes showed stronger associations between elevated GPR and increased epilepsy risk. No significant interactions were observed for surgery, smoking, drinking, or temporal lobe involvement.

Figure 5 presents subgroup analyses examining interaction effects between GPR and early PTE risk factors. Significant interactions were observed for sex (*P*-interaction = 0.05), hypertension (*P*-interaction = 0.029), and diabetes (*P*-interaction = 0.022). Gender difference demonstrated stronger associations between elevated GPR and epilepsy risk, while hypertensive patients and non-hypertensive showed more pronounced GPR effects on early epilepsy development. Diabetes also significantly modified the GPR-epilepsy association. No significant interactions were found for surgery, smoking, alcohol use, or temporal lobe involvement.

### 3.7 GPR group trend test

We performed a trend analysis using the three models constructed above to assess the association between GPR quartiles and Early PTE. Table 4 summarizes the results of this trend analysis. In the unadjusted model (Model-1), patients in the highest GPR quartile (Q4: 2.027–8.314) had a significantly increased risk of epilepsy compared to those in the lowest quartile (Q1: 0.441–1.311), with an OR of 4.016 (95% CI: 1.979–8.150, *P* < 0.001). This strong association remained significant in the partially adjusted model (Model-2), where Q4 showed an OR of 3.892 (95% CI: 1.884–8.043, *P* < 0.001).

**Table 4:** GPR group trend test.

**Exposure**	**Model-1**		**Model-2**		**Model-3**	
**GPR group**	**OR (95%CI)**	***P*** **-value**	**OR (95%CI)**	***P*** **-value**	**OR (95%CI)**	***P*** **-value**
Q1 (0.441–1.311)		1.000		1.000		1.000
Q2 (1.312–1.571)	1 (0.413, 2.423)	1.000	0.933 (0.384, 2.268)	0.878	0.711 (0.288, 1.757)	0.460
Q3 (1.572–2.026)	1.411 (0.621, 3.207)	0.411	1.336 (0.585, 3.052)	0.492	0.739 (0.310, 1.764)	0.496
Q4 (2.027–8.314)	4.016 (1.979, 8.150)	< 0.001	3.892 (1.884, 8.043)	< 0.001	1.594 (0.715, 3.555)	0.254
GPR group trend	3.149 (2.028, 4.891)	< 0.001	3.175 (1.999, 5.010)	< 0.001	1.840 (1.114, 3.039)	0.017

Model-1: non-adjusted.

Model-2: adjust for: Gender, Age, Smoking, Drinking, Hypertension, Diabetes.

Model-3: adjust for: GCS, Age, PLT, RBC, Lymphocyte, WBC, P, Globulin, Hypertension, Frontal_lobe, Temporal_lobe, Surgery.

GCS, Glasgow Coma Scale; PLT, Platelet; RBC, Red Blood Cell; WBC, White Blood Cell; P, Phosphorus; GPR, Glucose-Potassium Ratio; OR, Odds Ratio; CI, confidence interval.

However, in the Model-3, which accounted for demographic, clinical, and biochemical factors, the association for Q4 was no longer statistically significant (OR: 1.594, 95% CI: 0.715–3.555, *P* = 0.254). Despite this, the overall trend across GPR quartiles remained significant in all models, with increasing GPR levels associated with higher risk of Early PTE. The trend test yielded ORs of 3.149 (95% CI: 2.028–4.891, *P* < 0.001) in Model-1, 3.175 (95% CI: 1.999–5.010, *P* < 0.001) in Model-2, and 1.840 (95% CI: 1.114–3.039, *P* = 0.017) in Model-3.

## 4 Discussion

This study establishes the GPR as a robust and independent predictor of Early PTE in patients with TBI. Logistic regression analyses, both unadjusted and adjusted, demonstrated a significant association between elevated GPR and increased risk of Early PTE. RCS analysis revealed a non-linear relationship, identifying a critical threshold at GPR = 2.835. KM curve analysis further confirmed that TBI patients with GPR values exceeding this threshold exhibited a significantly higher incidence of Early PTE. Subgroup analyses further revealed that hypertension, diabetes, and gender influenced the association between GPR and early epilepsy. Underscoring the clinical relevance of this biomarker for risk stratification.

Previous studies by Zhou et al. established the GPR as a reliable predictor of 30-day mortality in severe TBI (5). Building upon this foundation, our findings further corroborate the growing of evidence supporting the prognostic significance of biochemical markers, particularly GPR, which serves as a composite index reflecting both immune-inflammatory status and cellular energy metabolism during physiological stress. Mechanistically, elevated GPR levels indicate a pro-inflammatory state coupled with impaired energy metabolism, creating an unfavorable internal environment that compromises recovery and contributes to poor clinical outcomes. This pathophysiological framework is further supported by Zhou et al.'s work demonstrating GPR's strong correlation with 6-month prognosis in spinal cord injury (13). Our study extends these clinical applications by establishing GPR's predictive value for Early PTE, complementing prior research by Wu et al. that highlighted GPR's association with broader neurological outcomes and its practical advantages as an accessible, cost-effective biomarker (14). Unlike previous investigations primarily examining mortality or general neurological deterioration, our work provides novel mechanistic insights into GPR's specific role in epileptogenesis. Notably, we identified a non-linear dose-response relationship with a critical threshold at GPR = 2.835—a finding that aligns with emerging evidence on non-linear metabolic-neurological interactions and offers potential clinical utility for risk stratification.

Subgroup analyses in our study align with previous clinical observations by Chen et al., who reported significantly higher incidence rates of early post-traumatic Epilepsy PTE in hypertensive patients compared to normotensive individuals (15, 16). This association is further supported by metabolic studies demonstrating that diabetes mellitus independently increases susceptibility to early PTE following TBI, as evidenced by Mahler et al.'s epidemiological findings (17). The pathophysiological basis for this relationship is reinforced by experimental work from Xia et al., where hyperglycemia directly induced epileptogenic activity in rodent models (18). Beyond metabolic factors, clinical TBI research has consistently identified biological sex as an independent predictor of Early PTE risk (15, 19). Our trend analysis substantiates these clinical observations by revealing a clear dose-response relationship between ascending GPR quartiles and escalating epilepsy risk. These collective findings not only validate GPR's reliability as an economical prognostic biomarker but also demonstrate its practical utility for early risk stratification in TBI populations. The convergence of clinical, experimental, and biochemical evidence positions GPR as a robust tool for identifying high-risk patients during the critical post-injury window.

The present research establishes the GPR as an independent predictive biomarker for Early PTE, with three principal mechanistic pathways identified: (1) Systemic Inflammatory Modulation: The composite GPR index reflects concurrent hyperglycemic and hypokalemic states that potentiate systemic inflammation post-TBI. Traumatic injury triggers substantial release of pro-inflammatory cytokines (IL-6, TNF-α, IL-1β) that induce stress hyperglycemia (20), which subsequently enhances neuronal excitability through glutamate-mediated mechanisms (21, 22). Paradoxically, these inflammatory mediators simultaneously suppress Na^+^-K^+^-ATPase activity and potassium channel function, creating a hypokalemic state (23). This dual pathology establishes GPR as a sensitive indicator of both systemic inflammation severity and consequent neuronal network destabilization. (2) Neuroinflammatory Cascades: Localized neuroinflammation following TBI involves microglial/astrocytic activation and cytokine-mediated ion channel dysfunction. Activated microglia release inflammatory factors that specifically disrupt potassium and chloride channel homeostasis, generating epileptiform activity (24). Elevated GPR compounds this pathology by exacerbating oxidative stress—ROS-mediated neuronal membrane damage activates NF-κB signaling, establishing a self-perpetuating cycle of inflammation and hyperexcitability through continued cytokine release (25, 26). (3) GPR and Immune Cell Function: Following TBI, disruption of the blood-brain barrier allows peripheral immune cells to infiltrate the central nervous system (27), and serving as a quantitative marker of this process. Hypokalemia impairs immunoregulatory T-cell function (28), while hyperglycemia promotes neutrophil/monocyte activation via the AGE-RAGE pathway (29). The resultant cytokine storm further disrupts neuronal networks, completing the triad of metabolic, inflammatory, and immunological disturbances that collectively lower seizure threshold and promote epileptogenesis.

## 5 Limitations

Despite its strengths, this study has several limitations that warrant consideration. These limitations highlight areas that require further investigation to strengthen the generalizability and applicability of our findings. (1) The study design was retrospective, which may introduce selection bias and limit the ability to establish causality between GPR and Early PTE. (2) The study was conducted in a single center, potentially limiting the generalizability of the findings to other populations or healthcare settings. (3) Although comprehensive adjustments for confounding factors were made, the possibility of residual confounding cannot be completely ruled out. (4) The study relied on routinely collected biochemical data, which might vary in quality and standardization across different institutions. (5) This study still has several independent risk factors in the logistic regression analysis, such as age, temporal lobe injury, and whether surgery was performed, which require further detailed analysis. Moreover, a multivariate logistic regression analysis is lacking. Additionally, as an independent risk factor, GPR has not been subjected to calibration curve Hosmer-Lemeshow (H-L) testing, nor has the clinical application efficacy of the decision curve analysis (DCA) been evaluated. (6) The lack of external validation in an independent cohort limits the robustness of the findings for wider clinical application.

## 6 Suggestions and perspectives

Future studies should adopt a prospective, multicenter design to validate the findings across diverse populations and settings. Incorporating additional biomarkers, such as inflammatory markers or neuroimaging data, could enhance the predictive power of the GPR model. Further exploration of the mechanisms linking GPR to epileptogenesis is needed, using experimental models to clarify the biological pathways involved. Developing risk prediction tools that integrate GPR with other clinical and biochemical parameters may improve risk stratification and personalized management of TBI patients. Longitudinal studies with extended follow-up periods are essential to evaluate the long-term predictive value of GPR for late-onset epilepsy.

## 7 Conclusions

This study underscores the potential of the GPR as a valuable biomarker for predicting Early PTE in TBI patients, offering a cost-effective and accessible tool for early risk stratification. However, further validation through prospective multicenter studies is essential to confirm its clinical utility and generalizability. Future research should exploring the underlying mechanisms linking GPR to epileptogenesis could also provide insights for developing targeted therapies, ultimately improving the management and outcomes of TBI patients at risk of Early PTE.

## Data Availability

The raw data supporting the conclusions of this article will be made available by the authors, without undue reservation.
